# Two cases of monomicrobial intraabdominal abscesses due to KPC - 3 *Klebsiella pneumoniae *ST258 clone

**DOI:** 10.1186/1471-230X-11-103

**Published:** 2011-09-30

**Authors:** Paola Di Carlo, Gianni Pantuso, Alessia Cusimano, Francesco D'Arpa, Anna Giammanco, Gaspare Gulotta, Adelfio M Latteri, Simona Madonia, Giuseppe Salamone, Caterina Mammina

**Affiliations:** 1 Department of Sciences for Health Promotion "G. D'Alessandro", University of Palermo, Via del Vespro 133, Palermo, I-90127, Italy; 2 Department of Surgery and Oncology, University of Palermo, Via del Vespro 133, Palermo, I-90127, Italy; 3 Department of General Surgery, Urgency and Organ Transplantation, University of Palermo, Via del Vespro 133, Palermo, I-90127, Italy

**Keywords:** monomicrobial abscess, *Klebsiellae pneumoniae*, carbapenemases

## Abstract

**Background:**

Knowledge of the etiology of pyogenic liver and pancreatic abscesses is an important factor in determining the success of combined surgical and antibiotic treatment. Literature shows geographical variations in the prevalence and distribution of causative organisms, and the spread of *Klebsiella pneumoniae *carbapenemase-producing bacteria is an emerging cause of abdominal infections.

**Case presentation:**

We herein describe two cases of intra-abdominal abscesses due to monomicrobial infection by *Klebsiella pneumoniae *Sequence Type 258 producing *K. pneumoniae *carbapenemase 3 (KPC-Kp). In case 1, a 50-year-old HIV-negative Italian woman with chronic pancreatitis showed infection of a pancreatic pseudocystic lesion caused by KPC-Kp. In case 2, a 64-year-old HIV- negative Italian woman with pancreatic neoplasm and liver metastases developed a liver abscess due to KPC after surgery. Both women were admitted to our hospital but to different surgical units. The clonal relationship between the two isolates was investigated by pulsed-field gel electrophoresis (PFGE). In case 2, the patient was already colonized at admission and inter-hospital transmission of the pathogen was presumed. A long-term combination regimen of colistin with tigecycline and percutaneous drainage resulted in full recovery and clearance of the multidrug-resistant (MDR) pathogen.

**Conclusions:**

Timely microbiological diagnosis, the combined use of new and old antibiotics and radiological intervention appeared to be valuable in managing these serious conditions. The emergence and dissemination of MDR organisms is posing an increasing challenge for physicians to develop new therapeutic strategies and control and prevention frameworks.

## Background

Pyogenic abdominal abscess caused by carbapenemase-producing Gram-negative pathogens is an emerging infection in surgical and intensive care settings. Failure to timely diagnose these infections and treat them appropriately has been associated with increased rates of therapeutic failure and mortality [1-3].

Multidrug resistant (MDR) pathogens in liver or pancreatic abscesses have been reported since 1986, but more recently MDR *Klebsiella pneumoniae *has been described as an emerging causative agent [4,5]. Moreover, a high incidence of septic metastatic lesions in patients with *Klebsiella pneumoniae *liver abscess has also been reported [6,7]. Consequently, early detection with the increasingly significant diagnostic contribution of computed tomography (CT), as well as a more accurate knowledge of the most likely causative pathogens and their antibacterial drug resistance patterns, are critical.

Post-operative abscesses may occur after traditional or laparoscopic surgery and other micro-invasive techniques [8-11]. Hypermucoviscosity, virulence genes such as rmpA (regulator of mucoid phenotype) and aerobactin (an iron siderophore) and capsular serotypes K1 and K2 have been identified such as important virulence determinants in Klebsiella pneumoniae for liver abscesses [12]. There are predisposing factors that increase the risk of developing an abscess, most commonly linked to bile duct obstruction, metabolic causes like diabetes ketoacidosis, and nosocomial acquisition [13]. Enteric organisms are more often able to pass through the portal venous system or its branches [14]. Thus, intestinal colonization with MDR organisms, such as carbapenem resistant *K. pneumoniae*, may be a risk factor in critically ill surgical patients. Post-surgical wound infections involving these organisms have also been described [15].

However, in some cases the source of infection may remain undetected and primary abscesses due to virulent clones of *K. pneumoniae *have been recently described in otherwise healthy carriers [13,16].

Carbapenem resistance in *K. pneumoniae *can be mediated by the production of carbapenemases belonging to Ambler class A, B or D. The most common carbapenemase worldwide is *K. pneumonia*e carbapenemase (KPC), an Ambler molecular class A enzyme that hydrolyses a broad variety of β-lactams [17]. In addition, KPC-producing isolates demonstrate resistance to many agents commonly used to treat Gram-negative infections, including quinolones and aminoglycosides. The globally distributed ST of *K. pneumoniae *associated with KPC enzyme production is ST258 [17]. However, despite the internationally prominent role of ST258, other *K. pneumoniae *STs reported as carrying KPC include STs 14, 21, 37, 45, 101, 228, 234, 257 and 259 in the United States, and STs 277, 327, 340 and 376 in Israel [18]. The potential for the spread of plasmid encoded KPC carbapenemases to different strains, including those locally more prevalent, is a serious cause of concern [17,18].

In this report, we describe the management of two epidemiologically related cases of intra-abdominal abscess caused by multidrug resistant *K. pneumoniae *carbapenemase (KPC) 3 - producing *K. pneumoniae *(Kp) Sequence Type (ST) 258 in two hospitalized surgical HIV- negative patients.

## Case Presentation

### Case 1

A 50 year-old woman of Italian origin and nationality was admitted to the general surgery and emergency unit of the "P. Giaccone" Teaching Hospital in Palermo, Italy, in April 2010, with severe abdominal pain and chills. She reported a three week history of abdominal discomfort and distension, anorexia and weight loss. Her medical history included chronic pancreatitis (CP) associated with alcohol abuse and heavy cigarette smoking.

Upper gastro-intestinal (GI) endoscopy and colonoscopy yielded normal findings. Relevant laboratory values were as follows: AST 86 U/L (normal range: 0-41 U/L), ALT 87 U/L (normal range: 0-37 U/L), serum amylase 134 U/L (normal range 53-123 U/L), lipase 356 U/L (normal range 10-150 U/L) and Bilirubin 1.8 mg/dl (normal range 0.2-1.3 mg/dl). Oral glucose tolerance test was normal and the patient tested negative for HBV, HCV and HIV.

**Figure 1:**
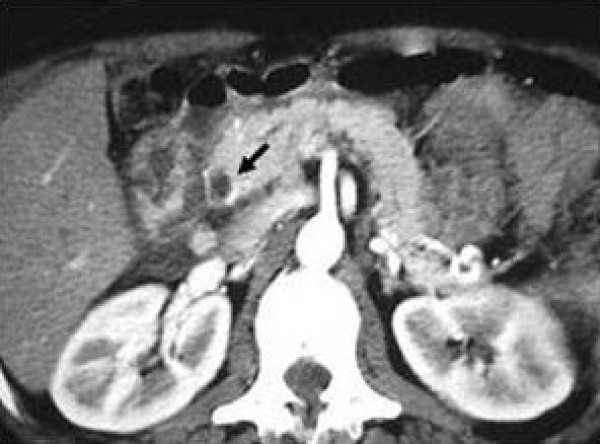
**Computed Tomography scan after four months in case 1**. Lesion found in the head of the pancreas (arrowhead).

On admission, a computed tomography (CT) scan showed a heterogeneous mass in the pancreatic head, while the rest of the organ appeared normal. The adjacent intra-pancreatic common bile duct and the pancreatic duct were normal. There was no evidence of vessel or lymph node involvement. The clinical suspicion was CP in the exacerbation phase. After conservative treatment, the patient's symptoms resolved and laboratory data returned to within the normal range. Four months later, the patient was re-admitted to the same hospital unit after developing fever with a WBC count of 16 × 10^9^/L (89% neutrophils, 7% lymphocytes, and 4% monocytes) and serum C-reactive protein levels (CRP) of 15 mg/dl (normal range 0.08-1.5 mg/dl). A CT scan of the abdomen and pelvis showed a relatively small (< 5 cm) pseudocystic lesion located in the head of the pancreas (Figure 1). Using a 21 gauge spinal needle, a CT scan-guided percutaneous aspiration biopsy was performed. A purulent fluid was aspirated and submitted for cytology and culture.

Cytology showed inflammatory cells without evidence of malignancy. Culture grew a multidrug-resistant isolate of KPC3-Kp ST258 isolate [19]. *bla*_KPC3 _gene was identified by polymerase chain reaction (PCR) and sequencing, as previously reported [19].

The carbapenem non-susceptible isolate was initially characterized by the Phoenix system (Becton Dickinson, Sparks, MD, USA) as resistant to amikacin and intermediately susceptible to gentamicin. Further antimicrobial-drug susceptibility testing using agar dilution and Etest (AB-Biodisk, Solna, Sweden) showed MICs in the susceptible range for tigecycline and colistin. The resistance pattern of the KPC-Kp isolate is shown in Table 1.

**Table 1:** Antimicrobial susceptibility patterns of *Klebsiella pneumoniae *isolates

Antimicrobial	Patient 1	Patient 2
	**MIC(μg/ml)**	

Amikacin	≥ 64	≥ 64

Ampicillin	≥32	≥32

Aztreonam	≥ 64	≥ 64

Cefazolin	≥64	≥64

Cefepime	32	32

Ceftriaxone	≥ 64	≥ 64

Ciprofloxacin	≥ 4	≥ 4

Gentamicin	8	8

Piperacillin-tazobactam	≥ 128	≥ 128

Tobramycin	≥ 6	≥ 6

Trimethoprim-sulfamethoxazole	≥ 320	≥ 320

Nitrofurantoin	256	256

Ertapenem	≥ 8	≥ 8

Meropemen	32	32

Imipenem	> 8	> 8

Moxifloxacin	> 8	> 8

Tigecycline*	0,5	0,5

Polymyxin B*	0.75	0.75

MIC, minimum inhibitory concentration. * Tested by Etest

Combined intravenous colistin (colistimethate sodium,1 mg of colistin equals 12,500 IU) at a dosage of 5 mg/kg/day divided in 3 equal doses and tigecycline (recommended dosage regimen 100 mg initially, followed by 50 mg every 12 hours) was started. After six weeks of combined antibiotic treatment, the symptoms resolved and leukocyte count, serum amylase and lipase returned within normal ranges. Instrumental assessment did not show any change in the size of the lesion throughout the observation period.

### Case 2

A 64-year-old HIV-negative woman of Italian origin and nationality was admitted in June 2010 to the surgical oncology unit of the same hospital with a three-month history of nausea, occasional vomiting and epigastric and right-side abdominal pain. On admission, a rectal swab was performed because she was enrolled in an ongoing one-year active surveillance program of multidrug resistant Gram negative colonization. Culture tested positive for a KPC3-Kp strain. Cohorting and contact isolation procedures were implemented. An aesophago-gastro-duodenoscopy (EGDS) tested negative for neoplastic infiltration and duodenal stenosis. A CT of the abdomen revealed a hypodense and inhomogeneous swelling of the pancreas head, compatible with a neoplasm, and a secondary lesion at the fifth liver segment. Therefore, a pancreatic-duodenectomy with resection of the fifth and the sixth liver segment was performed. A jejunostomy feeding tube and six abdominal drainages were also placed. Histology confirmed the diagnosis of intraductal papillary mucinous neoplasm of the pancreas with liver metastases.

**Figure 2:**
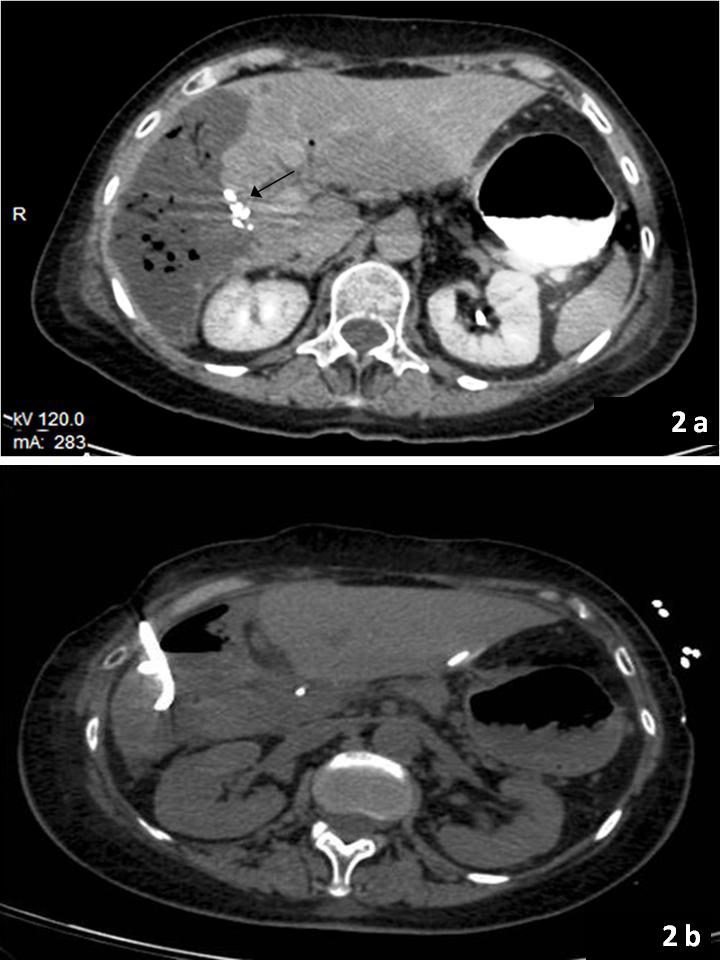
**Post-operative Computed Tomography scan in case 2**. a. Contrast-enhanced CT scan showed metal clips due to resection of liver segment V and VI (arrowhead) and infected lesion in VII and VIII liver segment (day 8). b. Percutaneous trans-hepatic drainage (day 10).

On the first post-operative day, the patient started enteral and parenteral nutrition. Her blood tests looked normal and she did well. On the seventh post-operative day, she developed fever, with laboratory tests revealing a WBC count of 19 × 10^9^/L and CRP of 13.6 mg/dl. An abdominal CT scan detected a liver lesion with air bubbles, consistent with an abscess in the right lobe segment (Figure 2a). Percutaneous trans-hepatic abscess drainage was performed (Figure 2b). Culture of the drainage fluid grew a multidrug-resistant ST258 KPC-Kp isolate. Antimicrobial susceptibility testing (Table 1) and molecular typing were carried out. The phenotypic and genetic make-up of this isolate was indistinguishable from that of the colonizing isolate.

Intravenous therapy with colistin and tigecycline using the dosage and administration procedure previously described for case 1 was started. During the first two weeks of antibiotic therapy combined with continuous percutaneous trans-hepatic drainage of the liver abscess, no clinical or instrumental improvement was observed. Repeated cultures of the drainage fluid consistently yielded Kpc-Kp. Only after five weeks of combined colistin and tigecycline treatment did the drainage culture test negative for KPC-Kp. The patient was eventually discharged after 45 days in hospital. She fully recovered, and on follow-up she proved to have returned to her usual state of health.

The clonal relationship between the isolates from the two patients was investigated by pulsed-field gel electrophoresis (PFGE). After digestion of total genomic DNA with restriction endonuclease *Xba*l, electrophoretic patterns were indistinguishable.

## Conclusions

The management of liver and pancreatic pyogenic abscesses is extremely complex. A treatment of choice that could be consistently adopted as a first line of intervention has still to be defined. The literature describes a wide range of approaches, ranging from simple conservative treatments associated with specific antibiotic therapy to surgical interventionist approaches using endoscopic or percutaneous drainage or the traditional open method [20-22]. However, while interventional endoscopy is a common strategy across recent reports, there is still a lot of debate over what is the best drainage technique to adopt [22,23].

Both patients described in this report underwent percutaneous drainage as there was no evidence of complications (e.g. intra-abdominal rupture, shock, peritonitis). This approach allowed us to diagnose the monomicrobial nature of the abscess. Although *K. pneumoniae *has previously been identified in western Europe as a causative agent of liver abscesses, monomicrobial infections, as appeared in both of our cases, have been rarely described [1,2].

When compared with other bacterial etiology, monomicrobial *K pneumoniae *liver abscesses are associated with a higher incidence of thrombophlebitis and septic hematogenous complications [6,7]. Therefore timely diagnosis, surgery and appropriate antimicrobial treatment are the mainstays to reduce mortality. Different surgical approaches have recently been suggested in patients with monomicrobial *K. pneumoniae *liver abscess (e.g. ultrasonography-guided percutaneous liver aspiration with retained catheter), whereas antimicrobial therapy strictly depends upon monitoring *in vitro *antimicrobial susceptibility using the clinical breakpoints defined by the European Committee on Antimicrobial Susceptibility Testing (EUCAST) [23,24].

The polymyxin class, known since 1947 and mostly represented by polymyxin B and polymyxin E (colistin), and tigecycline (the first representative of the new class of glycylcyclines) have recently been granted a major role in the treatment of the most problematic MDR Gram-negative pathogens (ESBL- and carbapenemase-producing strains) and enterobacteria. Previous studies have reported them being used as a monotherapy for patients with serious polymicrobial infections, including MDR microorganisms [25,26]. Both antibiotics show favorable penetration into various tissues, such as the sites of infection of the skin and soft tissues of the skin, as well as intra-abdominal infections (official indications). Apart from the ongoing effectiveness of new and old antibiotics, another crucial concern is whether a sufficient concentration of the antibiotic is able to reach the target site. It is known that penetration of an antibiotic into an encapsulated purulent lesion is likely to be limited and highly dependent on the degree of abscess maturation [27]. Tigecycline concentrations in peritoneal fluid have also been determined, but pharmacokinetic and pharmacokinetics and/or tissue penetration in intra-pancreatic abscesses is unknown [2,22].

In our experience, combination therapy appeared to be able to eradicate KPC-Kp. Though several reports have been published about infections caused by carbapenem resistant Gram negative pathogen in different clinical settings, no univocal data on the clinical efficacy of mono- or combined antimicrobial therapy in patients with liver or pancreatic abscesses are available, because their results are often included within those of patients with abdominal infection. In a review of current literature, Hirsch and Tam (2010), conclude that clinical success rates are low when polymyxins are used as monotherapy, but higher when they are used in combination with tigecycline or gentamicin [28]. Accordingly, combined tigecycline and colistin has shown good *in vitro *synergy against carbapenem resistant *Acinetobacter baumannii *with high level imipenem resistance [29]. Furthermore, it is a cause for great concern that colistin resistant ST258 KPC-Kp isolates have recently been detected in Europe, with an outbreak occurring in our geographical area also, while tigecycline resistance during therapy has already been observed in ICUs and other surgical critical care settings [19,25,30].

Future prospective studies are needed to answer important clinical questions, such as appropriate dosage and the most favorable pharmacokinetic/pharmacodynamic profiles for abscess penetration, while resistance development must be carefully monitored.

The KPC3-Kp isolates from the two patients were found to be indistinguishable by PFGE. An epidemiological relation between the two cases could not be excluded. However, clonal dissemination within healthcare structures of KPC-Kp ST258 has been described in many countries [17,18]. Moreover, an outbreak caused by a virtually indistinguishable strain was recently reported in another hospital in Palermo [19]. Hence, this does not allow us to draw reliable conclusions about the relationship between strains on the basis of their macrorestriction profiles.

A few studies have tried to explain the occurrence or portal of entry of MDR pathogens in surgical populations by involving the portal venous system or mesenteric lymph nodes [14,31]. In case 2, the patient was already colonized at admission and her clinical history was positive for previous hospitalization. In this case, intra-operative spread of colonizing KPC-Kp through the mentioned mechanisms can be hypothesized.

As a new diagnostic perspective, intra-operative bacterial translocation detection by bacterium-specific ribosomal RNA-targeted reverse-transcriptase PCR from lymph nodes has been described as able to predict post-operative infectious complications after major hepatic surgery whereas as new therapeutic perspective time-kill methodology has been described as able to investigate the in vitro interaction of antimicrobial combination [32].

Finally, strict infection control procedures according to the guidelines of the Centers for Disease Control and Prevention (CDC) for infection with carbapenem-resistant or carbapenemase-producing *Enterobacteriaceae *in acute care facilities are crucial in an era of crisis regarding the availability and effectiveness of antimicrobials [33].

In conclusion, two interesting findings emerge from these case reports. Firstly, adequate drainage along with an appropriate long term antibiotic therapy may lead to a favorable outcome in serious infectious events, such as liver and pancreatic abscesses. Secondly, the emergence and dissemination of MDR organisms is posing an increasing challenge for physicians to develop new therapeutic strategies and control and prevention frameworks.

## Consent

Written informed consent was obtained from the patients for publication of these case reports and accompanying images.

## List of abbreviations

ALT: alanine aminotransferase; AST: aspartate aminotransferase; MIC: minimum inhibitory concentration; WBC: white blood cell.

## Competing interests

The authors declare that they have no competing interests.

## Authors' contributions

PD designed the study and drafted the manuscript. GP and SM set up and implemented the study in the field and contributed to the interpretations of results. AML, GG, FD, GS and AC were responsible for the acquisition and interpretation of surgical data; AG provided microbiological data; CM supported molecular epidemiological results, supervised the study and revised the manuscript. All authors have read and approved the final manuscript.

## Pre-publication history

The pre-publication history for this paper can be accessed here:

http://www.biomedcentral.com/1471-230X/11/103/prepub
